# Single-photon frequency shifting with a quadrature phase-shift keying modulator

**DOI:** 10.1038/s41598-020-79511-8

**Published:** 2021-01-11

**Authors:** Changchen Chen, Jane E. Heyes, Jeffrey H. Shapiro, Franco N. C. Wong

**Affiliations:** Research Laboratory of Electronics, Massachusetts Institute of Technology, 77 Massachusetts Avenue, Cambridge, MA 02139 USA

**Keywords:** Applied optics, Optical physics, Other photonics

## Abstract

Deterministic frequency manipulation of single photons is an essential tool for quantum communications and quantum networks. We demonstrate a 15.65 GHz frequency shift for classical and nonclassical light using a commercially available quadrature phase-shift keying modulator. The measured spectrum of frequency-shifted single photons indicates a high carrier-to-sideband ratio of 30 dB. We illustrate our frequency shifter’s utility in quantum photonics by performing Hong–Ou–Mandel quantum interference between two photons whose initial frequency spectra overlap only partially, and showing visibility improvement from 62.7 to 89.1% after one of the photons undergoes a corrective frequency shift.

## Introduction

Photons are the carriers of choice for conveying quantum information over long distances to realize a scalable quantum internet owing to their weak interaction with the environment and their low-loss propagation in optical fiber^1,2^. Quantum information can be encoded in various photonic degrees of freedom, e.g., momentum, spatial mode, polarization, and frequency, which requires that these properties be precisely controlled. Although the momentum, spatial, and polarization modes of single-photon states can be easily manipulated with active or passive photonic components^3^, the control of frequency modes is less simple.

Spectral mode control is of particular interest in quantum networking. For example, the choice of optical wavelengths is often dictated by particular tasks such as low-loss long-distance transmission through optical fibers (telecommunication band around 1.55 $\upmu$m) or efficient interaction with atomic, ionic, or superconducting qubits. Fine frequency adjustments in the radio and microwave frequency ranges are just as important because fine tuning and matching the frequency spectra of photons from independent sources is required to ensure they produce high-fidelity quantum interference. For large frequency shifts on the order of terahertz or tens of terahertz, one relies heavily on nonlinear optical processes that work only at specific wavelengths and require strict phase-matching conditions^4–7^. Of interest in this work are small frequency shifts in the gigahertz to tens of gigahertz range. One method to deterministically shift a single photon’s frequency by $\omega _m$ is to apply a linear phase ramp $\phi (t) = \omega _m t$ over the entire wave packet of the photon, which can be realized using a fast electro-optic modulator^8^ or optomechanical waveguide^9^. However, because a phase ramp cannot be applied over an extended period of time (limited by the modulator’s maximum applied voltage), it is necessary to synchronize the timing of the phase ramp and the photon’s passage through the frequency-shifting device. This timing requirement therefore prevents the use of phase ramps on randomly generated photons such as those from continuous-wave (cw) pumped spontaneous parametric down-conversion (SPDC). Instead of a phase ramp, one can obtain frequency shifts based on optical single sideband (OSSB) modulation, which has been demonstrated for cw lasers using electro-optic modulators^10–13^. More recently, Lo and Takesue used a custom OSSB modulator to impose frequency shifts on single photons and they demonstrated its effectiveness by obtaining high visibility Hong–Ou–Mandel interference (HOMI) of two initially frequency-distinguishable SPDC photons^14^. For smaller frequency shifts of less than a gigahertz, it is also possible to use an acousto-optic modulator as an OSSB frequency shifter^15^.

In this paper, we report implementing the same OSSB approach of Lo and Takesue^14^ and achieving similar HOMI-visibility results by repurposing a commercially available quadrature phase-shift keying (QPSK) modulator as an OSSB frequency shifter. The availability of well-packaged commercial components allows for simpler and faster setup and typically offers better specifications than devices custom made for quantum photonics research. We characterized a commercial QPSK modulator for frequency shifting of single photons and compared spectra before and after the shift to show we realized a carrier-to-sideband ratio (CSR) of 30 dB. The frequency shifting technique also works well on a broadband source, showing high CSRs of $>20$ dB over its 2.4-THz spectrum. We observe significant improvement in HOMI visibility when we applied an appropriate frequency shift to one of two interfering SPDC photons whose initial frequency spectra overlapped only partially. Our frequency shifter based on a commercial QPSK modulator is thus a convenient and useful device for many quantum communication applications such as providing unconditional security for high-dimensional quantum key distribution system^16–19^ and making fine frequency adjustment to single photons for quantum information storage in solid-state quantum memories^20^. With additional intensity and phase control, the frequency shifter can also be used as a quantum pulse shaper for precise temporal and spectral mode tuning in a dense frequency-multiplexed quantum communication network^21^.

## Results

**Figure 1:**
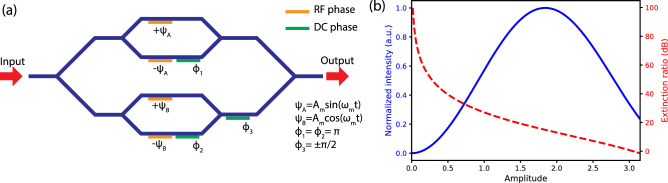
(**a**) Schematic of QPSK modulator with two radio-frequency (RF) phase modulators and three static phase shifters. All beam splitters and combiners are 50/50 coupled. (**b**) Shifted signal intensity (solid blue curve) and minimum extinction ratio (dashed red curve) as a function of driving amplitude $A_m$. The intensity of the shifted signal is normalized to the theoretical maximum value.

The QPSK modulator shown in Fig. 1a consists of two independent Mach-Zehnder interferometers embedded in a Mach-Zehnder interferometer superstructure. Optical inputs to the modulator must be linearly polarized with alignment along the *z*-axis of the modulator’s lithium niobate waveguide. The radio-frequency (RF) modulators in the top and bottom interferometers are driven by orthogonal RF signals $\Psi _A = A_m \sin \omega _m t$ and $\Psi _B = A_m \cos \omega _m t$, respectively, in a push-pull ($\pm \Psi _A$ and $\pm \Psi _B$) configuration, where $A_m$ is the RF signal amplitude and $\omega _m$ is the RF signal frequency. Using the Jacobi-Anger expansion, we can express these phase modulations as

1 $$\begin{aligned} e^{i A_m \sin \omega _m t} = \sum ^{\infty }_{k=-\infty } J_k (A_m) e^{ik\omega _m t},\;\; e^{i A_m \cos \omega _m t} = \sum ^{\infty }_{k=-\infty } i^k J_k (A_m) e^{ik\omega _m t}, \end{aligned}$$

where $J_k(A_m)$ is *k*-th Bessel function of the first kind. For an input field with amplitude $E_0$ and center frequency $\omega _0$, $E_{in}(t)=E_0 e^{i\omega _0 t}$, the output field from the QPSK modulator can be written in harmonics of the RF signal frequency $\omega _m$ as

2 $$\begin{aligned} E_{out}(t) = \frac{E_0}{4} e^{i\omega _0 t} \sum _{k=-\infty }^\infty J_k(A_m) e^{ik \omega _m t} \Big [ 1 + (-1)^k e^{i\phi _1} + \left( 1 + (-1)^k e^{i\phi _2}\right) i^k e^{i\phi _3} \Big ], \end{aligned}$$

where $\phi _1$, $\phi _2$, and $\phi _3$ are constant phase shifts imposed at the QPSK modulator, as indicated in Fig. 1a. To operate the QPSK modulator as a frequency shifter, we set $\phi _1 = \phi _2 = \pi$ and $\phi _3 = -\pi /2$ ($\pi /2$) for frequency blue (red) shift. For blue-shift operation, Eq. (2) simplifies to

3 $$\begin{aligned} E_{out}(t)= & \, \frac{E_0}{4} e^{i\omega _0 t} \sum ^{\infty }_{k=-\infty } J_k (A_m) e^{ik \omega _m t} \left( 1 + (-1)^{k+1} \right) \left( 1-i^{k+1} \right) \nonumber \\= &  \,E_{in}(t) \Big [ \dotsc + J_{-3}(A_m)e^{-i3\omega _m t} + J_{1}(A_m)e^{i \omega _m t} + J_{5}(A_m)e^{i 5\omega _m t} + \dotsc \Big ], \end{aligned}$$

the latter showing the three lowest non-zero harmonics. Equation (3) shows that the output carrier is frequency shifted from $\omega _0$ to $\omega _0 + \omega _m$ with amplitude $J_1(A_m)$, and that the two nearest sidebands have frequencies $\omega _0 - 3\omega _m$ and $\omega _0 + 5\omega _m$ with amplitudes $J_{-3}(A_m)$ and $J_5(A_m)$, respectively. We define the CSR for a specific sideband to be CSR$_k = |J_1(A_m)/J_k(A_m)|^2$ for $k\ne 1$. In Fig. 1b we plot the intensity of the frequency-shifted signal $|J_1(A_m)|^2$ (solid blue curve) and the minimum extinction ratio (dashed red curve), given by $\min _{k\ne 1}\mathrm{CSR}_k$, versus the RF signal amplitude $A_m$. Figure 1b shows clearly the competing effects of maximizing the frequency-shifted carrier signal and suppressing the sidebands as a function of RF signal strength $A_m$. At $A_m=1.8$, the frequency-shifted signal reaches its theoretical maximum and is 4.7 dB below the input intensity, if we exclude the system’s insertion and coupling losses. However, under this driving condition, the minimum extinction ratio is only 15 dB, which may not be sufficient for certain quantum applications as these sidebands act as noise sources that degrade quantum measurements. In order to further suppress the sidebands at undesirable frequencies, one needs to operate the modulator at lower RF amplitudes resulting in lower frequency-shifted signal strengths. For example, at $A_m = 0.85$, the shifted signal decreases to 44% of the theoretical maximum but the minimum extinction ratio increases to 30 dB.

### Measurements of the shifted spectra of cw laser light, frequency comb, and single photons

**Figure 2:**
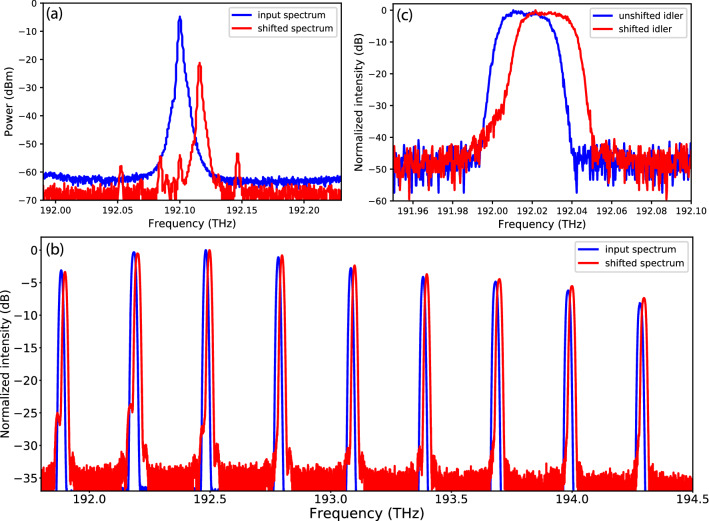
Input and output spectra for different input optical sources to the QPSK-modulator frequency shifter. (**a**) Spectra of the cw laser before and after the frequency shift. (**b**) Spectra of the frequency comb before and after the frequency shift, normalized to the maximum intensity. (**c**) Spectra of the idler photon before and after the frequency shift, normalized to the maximum detected signal-idler coincidence counts.

To characterize the performance of the QPSK modulator as a frequency shifter we first used a cw laser with sub-MHz linewidth at $\omega _0/2\pi$ = 192.1 THz (1560 nm wavelength) as its optical input. The RF modulation signal was set to perform an $\omega _m/2\pi$ = 15.65 GHz blue shift. Figure 2a shows the input and output spectra of the cw laser source for an RF amplitude $A_m = 0.57$, clearly showing the shift in frequency that we measured to be 16 GHz, limited by the 2-GHz resolution of our optical spectrum analyzer. The frequency shifted output power was measured to be 16.4 dB lower than the input power, a value that is accounted for by the expected drop in power, $|J_1(0.57)|^2 = -11.2$ dB, and the QPSK modulator’s 5.2 dB insertion loss. The choice of $A_m$ = 0.57 allows us to evaluate the CSRs. Figure 2a shows that there are 4 residual sidebands at $-3\omega _m$, $-\omega _m$, 0, and $3\omega _m$. The $-3\omega _m$ sideband is expected to have the largest amplitude according to Eq. (3) with CSR$_{-3}$ = 37 dB for $A_m = 0.57$ that matches the measured value. However, Eq. (3) also predicts that the sidebands at $-\omega _m$, 0, and $3\omega _m$ should have zero amplitude, whereas their non-zero peaks in Fig. 2a yield CSRs of $\sim$32 dB, which is the minimum extinction ratio of this measurement. The appearance of these three residual sidebands was likely caused by a slight difference in the splitting ratios of the 50/50 couplers within the QPSK modulator so that these sidebands did not completely cancel through destructive interference at the modulator’s output. Another nonideality of the QPSK modulator is that the static phase shifts $\phi _1$, $\phi _2$, and $\phi _3$ may drift due to thermal fluctuations in the phase shifters and cause the frequency shifting operation to degrade. In a separate measurement to check the stability of the QPSK modulator, we found the minimum extinction ratio decreased from 32 dB to 18 dB over 110 min due to phase drifts, and that the extinction ratio could be restored to 32 dB by adjusting the voltages of the phase shifters at the end of the 110-min duration.

Next we examined the frequency shifting operation for a broadband optical input to the QPSK modulator. From a broadband amplified spontaneous emission source we used a programmable intensity filter in the frequency domain to carve out a frequency comb spanning a $\sim$2.4 THz bandwidth. The frequency comb consisted of 20-GHz-wide comb teeth spaced at 300 GHz intervals. The RF driving conditions of the QPSK modulator were the same as those used for the cw laser input, and we optimized the setting to yield the highest minimum extinction ratio at optical frequency 193.4 THz (1550 nm wavelength). The spectra of the frequency comb before and after the QPSK-modulator frequency shifter are displayed in Fig. 2b, showing clearly that the entire spectrum was shifted by $\sim$16 GHz. We found that the minimum extinction ratio, optimized at 193.4 THz to yield 32 dB, decreased at frequencies lower than 193.4 THz, with the residual sideband peaks clearly visible in the lowest three comb lines at 191.9, 192.2, and 192.5 THz. The minimum extinction ratio for the 191.9 THz comb line was 22 dB. For the rest of the comb lines spanning more than 1.5 THz, the minimum extinction ratio of at least 30 dB was maintained. The measurements indicate that the QPSK modulator works well as a broadband optical frequency shifter but the extinction ratios can be uneven over a large bandwidth.

**Figure 3:**
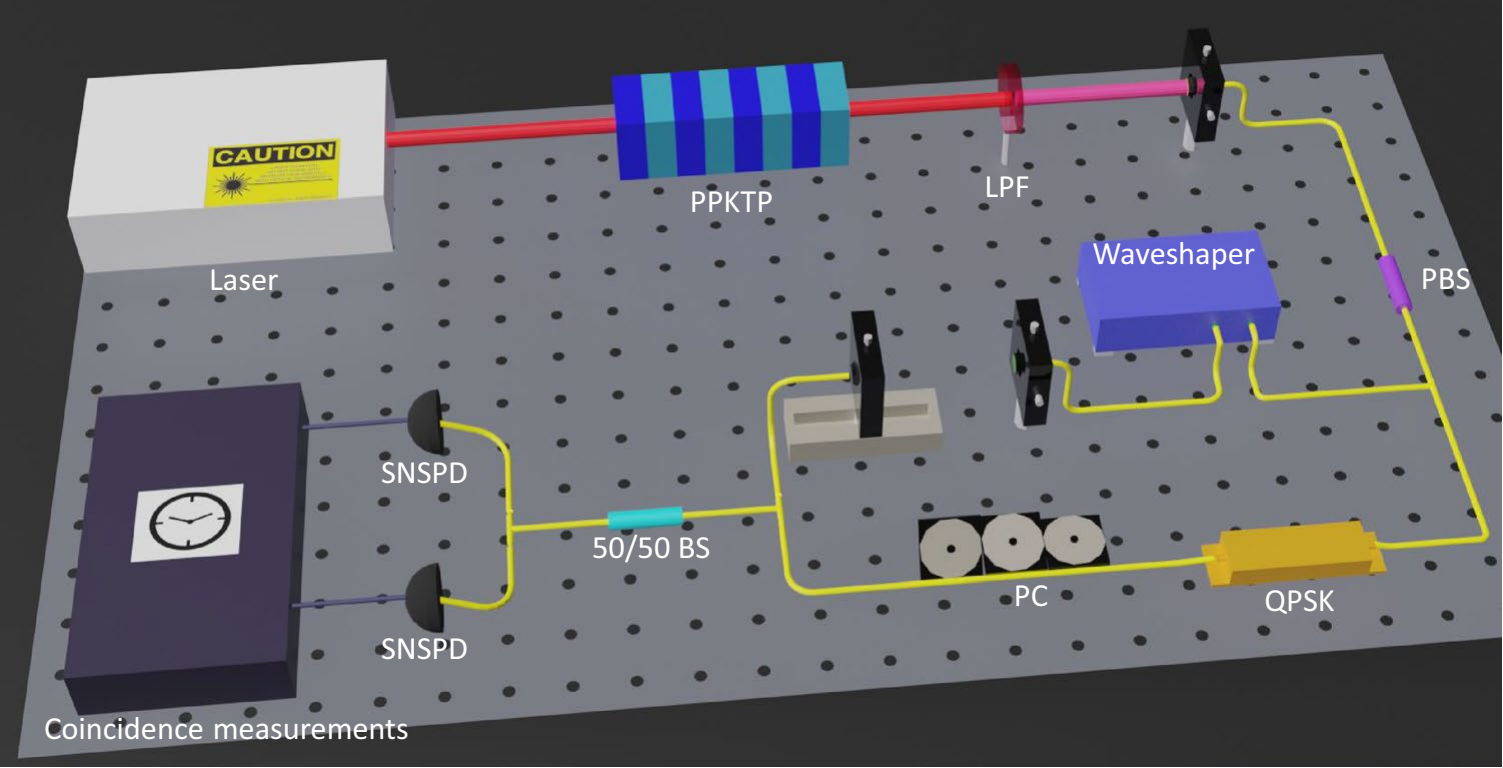
Schematic of the HOMI experiment. *PPKTP* periodically-poled potassium titanyl phosphate crystal, *LPF* long pass filter, *PBS* polarizing beam splitter, *QPSK* quadrature phase-shift keying modulator, *PC* polarization control, *50/50 BS* 50/50 beam splitter, *SNSPD* superconducting nanowire single-photon detector.

Additionally, we further examined the performance of the QPSK-modulator frequency shifter with heralded single photons as its optical input. From a type-II phase-matched periodically-poled potassium titanyl phosphate (PPKTP) waveguide SPDC source we generated signal and idler photon pairs whose center wavelength was $\sim$1560 nm. The orthogonally-polarized photons were separated by a polarizing beam splitter and sent to separate superconducting nanowire single-photon detectors (SNSPDs) with their arrival times recorded by a time tagger. Signal-photon detections were used to herald the presence of their idler-photon companions. We used the programmable intensity filter to implement a 50 GHz flat-top spectral filter on the signal photons that, by virtue of frequency entanglement, projected the corresponding idler photons to also have a 50 GHz spectrum centered at 192.014 THz. We sent the idler photons through a 10 ns/nm fiber Bragg-grating dispersion module for spectral analysis via time-to-frequency conversion^22^. Due to the imposed dispersion, the idler’s different spectral components propagated at different speeds resulting in different arrival times relative to the signal photon. Hence the idler spectrum can be obtained from the arrival time relative to that of the signal. In this particular measurement, we achieved a spectral resolution of 1.8 GHz, set by the Bragg-grating module’s spatial dispersion and the SNSPD’s timing jitter. Figure 2c shows the measured spectra of heralded idler photons before and after the frequency shift. The measured data are normalized to the maximum detected coincidence counts to remove the influence of the different transmission losses before and after measurements. The 15.65 GHz frequency shift of the single photon spectrum is clearly observed. The original spectrum is suppressed by 30 dB and is consistent with our measurement using the cw laser source as input in Fig. 2a. Our results demonstrate the ability to deterministically modify the spectral components of nonclassical light using a commercial QPSK modulator configured as a frequency shifter.

### Hong–Ou–Mandel interference of photon pairs

Hong–Ou–Mandel interference^23^, which measures the indistinguishability of two single photons, is an essential measurement tool in quantum photonics making the ability to fine tune the frequency of single photons to best match their spectra highly desirable. Figure 3 is the schematic of our HOMI measurement that utilized corrective frequency shifting to recover HOMI visibility by making the two interfering photons spectrally indistinguishable. The photon pairs were generated by the same cw SPDC source used for Fig. 2c’s single-photon frequency-shifting measurements. After the orthogonally-polarized signal and idler were separated, the signal photon was shaped by the programmable intensity filter to have a 50-GHz flat-top spectrum with a tunable center frequency. The corresponding idler, which also possessed a 50-GHz spectrum, went through the QPSK-modulator frequency shifter before recombining with the signal photon for HOMI coincidence measurements detected with two SNSPDs and then time tagged.

**Figure 4:**
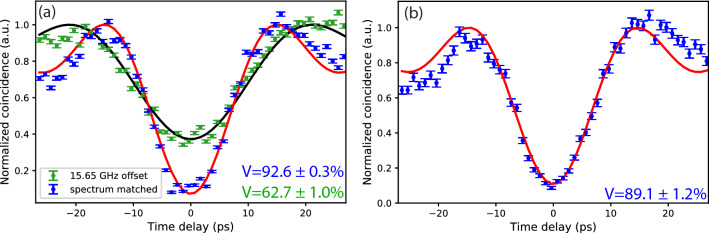
Hong–Ou–Mandel interference measurement results. (**a**) Normalized coincidence counts when the signal and idler had the same center frequency and matched spectra (blue) and when the signal and the blue-shifted idler spectra were offset by 15.65 GHz (green). The fitted functions for the matched and frequency-offset spectra are shown in red and black, respectively. (**b**) Normalized coincidence counts when the signal frequency was increased by 8 GHz so that it and the blue-shifted idler had nearly identical center frequencies. In both (**a**,**b**) the error bars mark the ±1 standard deviations of the detected coincidences’ Poisson noise and the maximum coincidence rates of the fitted functions are normalized to one. The background coincidence counts were measured and subtracted for each of the three measurements.

We first established the baseline visibility without frequency shifting, which denotes the maximum visibility achievable with our SPDC source. We tuned the center frequency of the signal spectral filter so that the filtered signal and the projected idler were frequency degenerate with overlapping spectra and therefore frequency indistinguishable. We measured the signal-idler coincidences as we varied the relative arrival-time delay between the signal and idler using an adjustable air gap in the signal path, as shown in Fig. 3. The HOMI measurement result without frequency shifting is shown in Fig. 4a (blue markers), fitted with a sinc function (red curve), from which we obtained a 92.6$\pm 0.3\%$ background-subtracted interference visibility. The HOMI visibility is defined as $(N_{max}-N_{min})/N_{max}$, where $N_{max(min)}$ is the maximum (minimum) detected coincidence count, and the uncertainty is calculated assuming Poisson noise. We calculated the background coincidence counts from accidental coincidences measured when the signal path but not the idler path to the 50/50 beam splitter was blocked, and vice versa. The background coincidence counts were primarily due to polarization leakage of the fiber polarization beam splitter.

We then blue shifted the idler by 15.65 GHz while the signal frequency remained unchanged, so that their center frequencies were now separated by the applied frequency shift. Because the signal and idler spectra were no longer entirely overlapping, the background-subtracted HOMI visibility decreased to 62.7$\pm 1.0\%$, as shown in Fig. 4a (green markers with black fitted curve, $\Delta \omega /2\pi =15.65$ GHz). To recover the HOMI visibility, the spectra of the filtered signal and the blue-shifted idler need to have the same center frequencies. We tuned the intensity filter to increase the signal’s center frequency by 8 GHz, which is about half of the applied frequency shift of 15.65 GHz. By energy conservation the sum frequency of the SPDC signal and idler equals the cw pump frequency. As a result, the 8 GHz increase in signal frequency reduced the idler frequency by 8 GHz so that their center frequencies became nearly identical. Note that we could not blue shift the signal by the exact 7.825 GHz amount (half of the RF frequency) owing to the programmable intensity filter’s minimum frequency-tuning step being too large. However, the 0.35 GHz difference in their center frequencies is relatively small compared with their 50 GHz bandwidth, thus any resultant degradation to HOMI is expected to be small. We remeasured the HOMI visibility, as shown in Fig. 4b (blue markers with red fitted curve), yielding a measured background-subtracted visibility of $89.1\pm 1.2\%$, close to the baseline visibility. We can interpret this measurement as starting with initial signal and idler center frequencies being separated by 16 GHz and a corrective frequency shift being applied to recover the frequency indistinguishability that is required for obtaining high HOMI visibility. We should remark that high HOMI visibility requires all degrees of freedom to match well between the two photons, suggesting that the QPSK-modulator frequency shifter did not materially affect other photonic degrees of freedom in this measurement. We note that the frequency shifter requires a linearly-polarized input with an output of the same polarization.

## Discussion

In this paper, we showed that we can operate a commercially available QPSK modulator as a frequency shifter. We demonstrated a 15.65 GHz deterministic frequency shift on narrowband and broadband classical light as well as on single photons. We showed the trade-off relation between the amplitude of the shifted signal and the minimum extinction ratio. At a 0.57 drive amplitude, we measured the noise sidebands to be at least 30 dB below the frequency-shifted signal. Furthermore, our measurement suggests that the frequency shift can be applied over a broadband range of $>1.5$ THz while maintaining a 30 dB extinction ratio. The amount of frequency shift is set by the applied RF signal frequency and is limited by the bandwidth of the QPSK modulator. Both high bandwidth RF sources and QPSK modulators are commercially available at up to 64 GHz, and they can be used to increase the frequency shift beyond what we have demonstrated. An even higher bandwidth electro-optical phase modulator at 100 GHz has also been reported^24^, suggesting future improvements as these devices become commercial. Additionally, we showed that this frequency shifting process only transforms the input photons in the frequency domain while preserving their original state in other degrees of freedom. We believe that QPSK-modulator frequency shifters will be useful in quantum communication and quantum network applications where frequency manipulation is desirable. The waveguide structure of QPSK modulators is compatible with photonic integration processes for more compact device integration with quantum sources and detectors.

## Methods

The QPSK modulator we used was a Fujitsu model FTM7961EX, with a 3 dB RF bandwidth of 22 GHz and a $V_\pi$ of less than 3.5 V. Its specified operational optical-wavelength range is from 1530 to 1610 nm, and we measured an optical insertion loss of 5.2 dB. The modulator can only transmit a linearly-polarized input aligned to the designed polarization for modulation in the lithium niobate waveguide. The 15.65 GHz RF signal was derived from an RF frequency synthesizer, amplified and split by a 0$^{\circ }$-phase 50/50 power splitter to serve as inputs to the QPSK modulator’s top and bottom interferometers. The two cables connecting the two splitter outputs to the modulator had a 10.96 cm length difference, corresponding to a $\pi /2$ phase shift, that was chosen to ensure orthogonality of the two driving RF signals, $\Psi _A$ and $\Psi _B$, as indicated in Fig. 1a. We also set the static phase shifts to $\phi _1 = \phi _2 = \pi$ and $\phi _3 = -\pi /2$ for a blue frequency shift.

The programmable intensity filter was a Finisar model 1000S waveshaper. This waveshaper provides control of the transmitted intensity from 0 dB to 50 dB attenuation at 1 GHz resolution over the entire telecommunication C band. The minimum optical bandwidth of the waveshaper was measured to be 12 GHz, limited by the point-spread function of the waveshaper’s optics, and we measured its insertion loss to be 5 dB. The fiber Bragg grating based dispersion module was manufactured by Proximion and imposes a dispersion amount of 10 ns/nm for inputs from 1558.58 nm to 1562.23 nm. We used the arrival times of idler photons at 1562.23 nm as a reference to convert the photons’ arrival times to frequencies.

Frequency-entangled photon pairs centered around 1560 nm were generated in a type-II phase-matched PPKTP waveguide^25^ pumped by a 780 nm cw laser. The signal and idler were partially overlapping in frequency and had a spectral full-width at half maximum bandwidth of 320 GHz. The signal and idler wave packets were time coincident within 2 ps. After the pump was filtered by a long-pass filter (Semrock BLP02-1319R-25), the orthogonally-polarized signal and idler were coupled into a polarization-maintaining (PM) fiber. In the HOMI experiment, the signal and idler were separated using a polarizing beam splitter. The signal was sent to the programmable waveshaper that functioned as a tunable 50 GHz flat-top spectral filter. After spectral filtering, the signal and idler can have the same or different spectra. The 50 GHz filter bandwidth was chosen so that it was greater than the waveshaper’s minimum optical bandwidth. The idler was sent to the frequency shifter before recombining with the filtered signal at a 50/50 beam splitter. The polarizations of the signal and idler were made to be the same before the beam splitter and their relative arrival-time delay at the beam splitter was controlled by a tunable air gap on a translation stage. Our niobium nitride (NbN) SNSPDs have 60% system efficiency, 150 ps timing jitter, < 400/s dark counts per detector, and 100 ns recovery time. The operational temperature for the SNSPDs was $\sim$0.8 K. The typical coincidence count in HOMI measurements was $\sim$200/s.

## Data Availability

All data regarding the work presented are available upon reasonable request to the corresponding author.
